# GITR Promotes the Polarization of TFH-Like Cells in *Helicobacter pylori*-Positive Gastritis

**DOI:** 10.3389/fimmu.2021.736269

**Published:** 2021-09-10

**Authors:** Siqi Ming, Huan Yin, Xingyu Li, Sitang Gong, Guoliang Zhang, Yongjian Wu

**Affiliations:** ^1^National Clinical Research Center for Infectious Diseases, Shenzhen Third People’s Hospital, Southern University of Science and Technology, Shenzhen, China; ^2^Center for Infection and Immunity, the Fifth Affiliated Hospital of Sun Yat-sen University, Zhuhai, China; ^3^Department of Gastroenterology, Guangzhou Institute of Pediatrics, Guangzhou Women and Children’s Medical Center, Guangzhou Medical University, Guangzhou, China

**Keywords:** TFH-like cells, *H. pylori*, gastritis, IL-21, GITR

## Abstract

Gastric CD4^+^T cells contribute to *Helicobacter pylori* (*H. pylori*)-induced gastritis by amplifying mucosal inflammation and exacerbating mucosal injuries. However, the pathogenic CD4^+^ T cell subset involved in gastritis and the potential regulators are still unclear. Here we identified an IL-21-producing gastric CD4^+^T cell subset, which exhibited tissue-resident CXCR5^−^BTLA^−^PD-1^hi^ TFH-like phenotype in *H. pylori*-positive gastritis patients. Meanwhile, we identified glucocorticoid-induced tumor necrosis factor receptor (GITR) as an important regulator to facilitate IL-21 production by CD4^+^T cells and accelerate mucosal inflammation in gastritis patients with *H. pylori* infection. Moreover, GITR expression was increased in gastric CD4^+^T cells of gastritis patients compared to healthy controls, along with the upregulated expression of its ligand GITRL in mucosal macrophages (Mϕ) of gastritis patients. Further observations showed that the activation of GITR/GITRL signal promoted the IL-21 production of CD4^+^T cells *via* the STAT3 pathway. Besides this, IL-21 from CD4^+^T cells induced the proliferation of B cell and promoted the production of inflammatory cytokines IL-1β and IL-6 and chemokines MIP-3α and CCL-25 as well as matrix metalloproteinase (MMP)-3 and MMP-9 by human gastric epithelial cells, suggesting the facilitating effect of IL-21-producing CD4^+^T cells on mucosal inflammation and injuries. Taking these data together, we revealed that GITR/GITRL signal promoted the polarization of mucosal IL-21-producing CD4^+^T cells in *H. pylori*-positive gastritis, which may provide therapeutic strategies for the clinical treatment of *H. pylori*-induced gastritis.

## Introduction

The interaction between invading pathogens and host immune factors could lead to the formation of an immune microenvironment that fosters chronic inflammation (1, 2). *Helicobacter pylori* (*H. pylori*)-induced gastritis usually results in mucosal injuries and extensive immune cell infiltration (3). Meanwhile, the inflammatory state in the mucosa could, in turn, impact the biological behavior of *H. pylori* (3). We previously demonstrated that Th9-like MAIT cells are enriched in gastric tissue and revealed the positive correlation of Th9-producing MAIT cells with disease progression (4). We also reported that *H. pylori*-associated antigens induced the Th1 and Th17 response of gastric CD4^+^ T cells (5). These observations indicate the tight interactions between *H. pylori* and the different subsets of immune cells and elements in the mucosal tissue, which may contribute to inflammatory response and mucosal pathology. Therefore, to fully understand the immunopathogenesis of inflammation in *H. pylori*-positive gastritis, it is critical to elucidate the interaction networks between inflammatory regulators and immune cells within the gastric mucosa.

T follicular helper (TFH) cell is a special CD4^+^T cell subset with the ability to induce B cell activation and differentiation (6). In secondary lymphoid tissues, TFH cells take part in the formation of the germinal center (GC) and regulate the development of T cell-dependent B cell responses (6). The unique feature of TFH is the high expression of C-X-C motif chemokine receptor 5 (CXCR5), inducible T cell co-stimulator (ICOS), and programmed cell death protein 1 (PD-1) as well as the nuclear transcriptional repressor B cell lymphoma 6 (BCL-6) (7). IL-21, mainly produced by TFH cells, performs a multifunctional role in a broad range of cells. IL-21 receptor (IL-21R) is expressed by a variety of cell types, including myeloid cells, natural killer (NK) cells, B cells, T cells, and non-immune cells (8). IL-21/IL-21R signal could regulate the generation and polarization of B cells and T cells and profoundly impact the function of Th cells, plasma cells, plasmablasts, and cells from GC (9–11). Furthermore, IL-21R could also be detected on non-immune cells, including thyroid cells (12), synovial fibroblasts (13), gastrointestinal fibroblasts (14), and gut epithelial cells (15), suggesting the possibly regulatory role of IL-21 for these non-immune cells.

Except for the roles that we introduced above, IL-21 also plays a key role in gastrointestinal mucosal immunity. IL-21expression was higher at the mucosal site of Crohn’s disease (CD) patients compared to ulcerative colitis (UC) patients and controls (16). The neutralization of IL-21 reduced the IFN-γ production in lamina propria T cells, which indicates that IL-21 may promote Th1 cell response (16). Furthermore, IL-21 prevents the TGF-β-dependent polarization of regulatory T (Treg) cells and promotes the differentiation of Th17 cells in experimental colitis (17). Moreover, IL-21 is highly produced in the gastric mucosa of *H. pylori*-infected patients (18) and could maintain the pro-inflammatory T cell immune response to drive chronic gastritis during *H. pylori* infection (19). However, the regulatory mechanism of IL-21-producing T cells in *H. pylori*-infected gastritis remains unclear.

The function of TFH cells is regulated simultaneously by the T cell antigen receptor binding strength (20) and the signaling transmission of co-stimulatory receptors (21), for example, CD28 exerts the important function in the early stage of TFH generation, while ICOS is critical in the later phases for TFH cell differentiation (21). In addition, the differentiation of TFH cell is dictated by the balance of cytokines, for example, IL-7 represses TFH cell formation by inhibiting BCL6 and CXCR5 expression (22). IL-12 produced by dendritic cells (DCs) induces the differentiation of IL-21-producing TFH-like cells (23).

Glucocorticoid-induced tumor necrosis factor receptor (GITR) is a member of tumor necrosis factor receptor superfamily (TNFRSF), which is constitutively expressed on T_reg_ cells with high levels and at low levels on unstimulated macrophages, T cells, and B cells (24). The trigger of GITR signal could promote effector T cell activation and reverse the suppressive function of T_reg_ cells (25). GITR also influences the progress of gastrointestinal disease by regulating mucosal immunity (26–29). Santucci et al. reported that GITR promotes the development of experimental colitis through enhancing the activation of macrophages and effector T cells (26). Liao et al. reported that GITR does not directly regulate T_reg_ and T effector cells but appears to promote the development of DCs and monocytes to enhance Th1 cell response, thus aggravating chronic enterocolitis (27). However, Uraushihara reported that GITR-expressing CD4^+^ T cells negatively regulate the intestinal inflammation and mucosal immune responses in experimental colitis (28). Besides this, Sakurai et al. reported that GITR negatively regulates intestinal inflammation by suppressing the IL-15-dependent NK cell activity (29). These studies indicate that GITR exhibits a controversial role in intestinal inflammation.

The role of GITR in *H. pylori*-infected gastritis remains unclear. Here we revealed the role of IL-21-producing TFH-like cells in *H. pylori*-positive gastritis. We identified a class of TFH-like CD4^+^ T cells with the capacity to secrete IL-21 in the gastric mucosa of *H. pylori*-positive gastritis patients and demonstrated that the GITR/GITRL axis promoted IL-21^+^CD4^+^ T cell polarization *via* the STAT3 signal pathway. Moreover, gastric CD4^+^T cell-producing IL-21 induced the proliferation of gastric B cells and the pro-inflammatory cytokines and chemokines as well as the secretion of MMPs by gastric epithelial cells. Collectively, our findings expand the understanding of IL-21^+^TFH-like CD4^+^ T cells in *H. pylori*-positive gastritis and investigate the underlying regulatory mechanism, which may provide potential interventions in the clinical treatment of *H. pylori*-induced gastritis.

## Results

### Increased IL-21-Producing TFH-Like CD4^+^ T Cells Are Observed in *H. pylori*-Positive Gastritis

**Figure 1:**
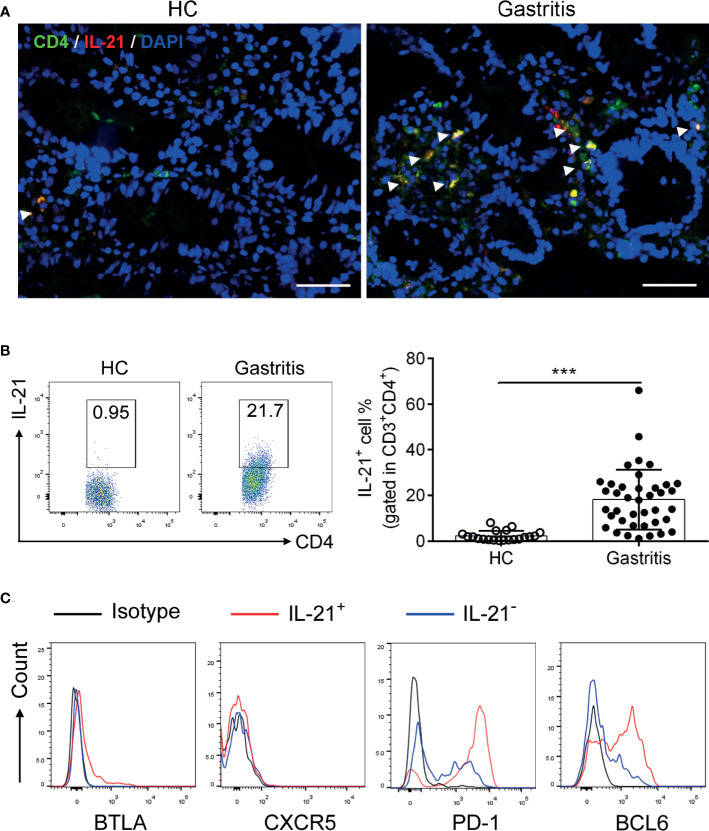
Identification of IL-21^+^TFH-like cells in *Helicobacter pylori*-positive gastritis. The biopsy specimens of gastric mucosa from *H. pylori*-positive patients (*n* = 39) and healthy controls (*n* = 20) were collected. **(A)** Immunofluorescence was conducted to determine the co-localization of CD4 (green) with IL-21 (red). The nucleus was stained with DAPI (blue). Scale bar: 100 µm. **(B)** The percentage of IL-21^+^CD4^+^ T cells in the gastric mucosa was detected by flow cytometry. **(C)** The expression of BTLA, CXCR5, PD-1, and BCL6 in IL-21^+^ or IL-21^-^ CD4^+^ T cells was assessed by flow cytometry. Unpaired Student’s *t*-test was performed to determine the difference between two groups. Data represent mean ± SD from at least three independent experiments. ***P < 0.001.

To explore the expression pattern of IL-21 in *H. pylori*-positive gastritis, we firstly collected biopsy samples from *H. pylori-*positive gastritis patients and healthy controls (the information of the participants is shown in **Table 1**) and detected the IL-21 level. The results showed that the co-localization of IL-21 with CD4^+^ T cells in the gastric mucosa was increased in *H. pylori*-positive gastritis patients (**Figure 1A**). In addition, an increased percentage of IL-21^+^ CD4^+^T cells was detected in biopsy samples from *H. pylori*-positive gastritis patients (**Figure 1B**). To identify the phenotype of IL-21^+^CD4^+^T cells, we analyzed the expression of TFH surface markers BTLA, CXCR5, and PD-1 as well as transcription factor BCL6. Interestingly, the gastric IL21^+^CD4^+^ T cells displayed a distinctive CXCR5^-^BTLA^-^PD-1^hi^ tissue-resident activated phenotype compared to IL21^-^CD4^+^ T cells (**Figure 1C**), whereas classical TFH cells were characterized as CXCR5^+^PD-1^+^BTLA^+^ (7). Meanwhile, these IL-21^+^ CD4^+^ T cells expressed a higher level of TFH-specific transcription factor BCL6 than IL21^-^CD4^+^ T cells (**Figure 1C**). Taking these data together, we found that IL-21^+^ CD4^+^T cells were increased and exhibited TFH-like phenotype in *H. pylori*-positive gastritis patients compared to healthy controls.

**Table 1:** Characteristics of healthy controls and gastritis patients.

	Healthy	Gastritis	*P-*value
Sample size (number)	20	39	–
Age, years (mean ± SD)	29.44 ± 12.65	31.35 ± 15.64	0.457
Sex (M/F)	11/9	17/22	0.426
Indication for endoscopy (%)			
Recurrent abdominal pain	NA	25 (43.8)	<0.001***
Burning abdominal discomfort	NA	21 (53.8)	<0.001***
Acid reflux symptoms	NA	19 (48.7)	<0.001***
Dyspepsia	NA	22 (56.4)	<0.001***
Epigastric pain	NA	29 (74.4)	<0.001***
Endoscopic finding (%)			
Normal	20 (100)	0 (100)	<0.001***
Gastritis	0 (0)	39 (100)	<0.001***
^13^C-urea breath test positive (DOB >5) (%)	0 (0)	39 (100)	<0.001***
*H. pylori* infection (%)	0 (0)	39 (100)	<0.001***

The level of significance is evaluated by unpaired Student’s t-test or chi-square test. P-value < 0.05 is considered significant.

F, female; M, male; DOB, delta over baseline; NA, no application.

***P < 0.0001.

### GITR Promotes IL-21 Production of Mucosal TFH-Like Cell Polarization in *H. pylori*-Positive Gastritis

**Figure 2:**
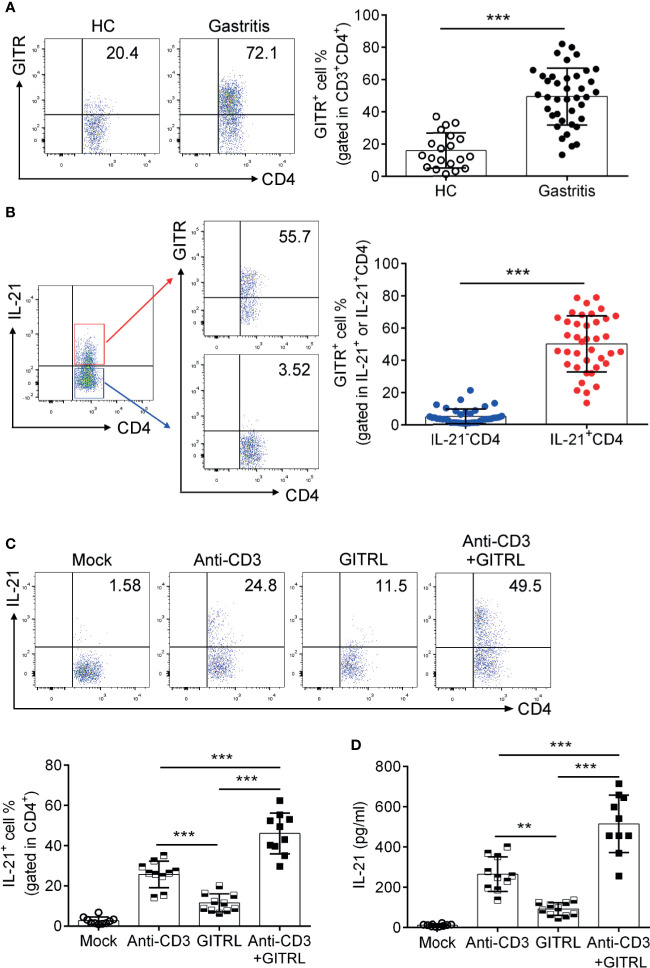
Glucocorticoid-induced tumor necrosis factor receptor (GITR) promoted the polarization of 21^+^TFH-like cells in *Helicobacter pylori*-positive gastritis. Biopsy samples of gastric mucosa from *H. pylori*-positive patients (*n* = 39) and healthy controls (*n* = 20) were collected. The GITR expression in **(A)** CD4^+^T cells and **(B)** IL-21^+^ or IL-21^-^CD4^+^T cells was determined by flow cytometry. **(C)** The sorted gastric CD4^+^T cells were stimulated with CD3 agonist Ab or recombinant GITRL protein. At 3 days later, IL-21 production by CD4^+^T cells was determined by flow cytometry. **(D)** CD4^+^T cells were isolated from the gastric mucosa of *H. pylori*-positive patients and treated with CD3 agonist Ab or recombinant GITRL protein. At 3 days later, the IL-21 concentration in the culture supernatant was determined by ELISA. Unpaired Student’s *t*-test was performed to determine the difference between the two groups. One-way ANOVA was used to compare among three or multiple groups. Data are displayed as mean ± SD from at least three independent experiments. **P < 0.01; ***P < 0.001.

GITR has been reported to be related to the differentiation of TFH cells (30). To explore whether GITR facilitated IL-21 production by TFH-like cells in *H. pylori*-positive gastritis, we determined the alternation of IL-21 production after GITR intervention. We firstly analyzed the GITR expression in *H. pylori*-positive gastritis. Consistent with the increased percentage of IL-21-producing TFH-like cells, the expression of GITR was also elevated in mucosal CD4^+^T cells from *H. pylori*-positive gastritis patients (**Figure 2A**). A further analysis indicated that IL-21^+^CD4^+^T cells exhibited a higher GITR expression compared to IL-21^-^CD4^+^T cells (**Figure 2B**). To determine the effect of the GITR/GITRL axis on IL-21 production of FTH-like CD4^+^T cells, mucosal CD4^+^T cells were sorted from *H. pylori*-positive gastritis patients and treated with recombinant GITRL protein to activate the GITR on CD4^+^T cells. After the treatment of mucosal CD4^+^T cells with recombinant GITRL, an increased percentage of IL-21^+^CD4^+^T cells was detected (**Figures 2C, D**). Moreover, the combination of CD3-activating antibody (Ab) with recombinant GITRL further enhanced the IL-21 production of CD4^+^T cells (**Figures 2C, D**). Based on the abovementioned results, we demonstrated that GITR promoted the IL-21 production of mucosal CD4^+^T cells in *H. pylori*-positive gastritis.

### GITRL Is Upregulated in Mucosal Macrophage and Provides a Ligand Signal to TFH-Like Cells

**Figure 3:**
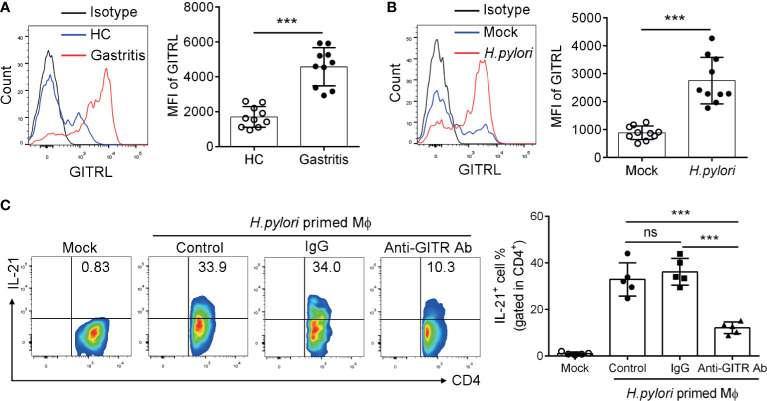
GITRL was upregulated in macrophage and provided ligand signal to TFH-like cells in *Helicobacter pylori*-positive gastritis. **(A)** GITRL expression was determined in the mucosal macrophage of *H. pylori*-positive patients (*n* = 10) and healthy controls (*n* = 10) by flow cytometry. **(B)** Monocyte-derived macrophages (MDM) were infected with *H. pylori* (multiplicity of infection, MOI = 20) for 24 h. GITRL expression was assessed by flow cytometry. **(C)** MDM was infected with *H. pylori* (MOI = 20) and co-cultured with gastric CD4^+^T cells in the presence of control IgG or anti-GITR-neutralizing Ab. At 3 days later, IL-21 production by CD4^+^T cells was detected. Unpaired Student’s *t*-test was performed to determine the difference between the two groups. One-way ANOVA was used to compare among three or multiple groups. Data are shown as mean ± SD from at least three independent experiments. ns, *P* > 0.05; ***P < 0.001.

Next, to investigate the effect of the GITRL/GITR axis on the function of TFH-like cells, we determined the expression and the modulatory role of GITRL in *H. pylori*-positive gastritis patients. GITR is primarily expressed by T cells, and its ligand GITRL is predominantly expressed on myeloid-derived cells, in particular on macrophage (Mϕ). We then detected the expression of GITRL on mucosal Mϕф(CD11b^+^ HLA-DR^+^ CD68^+^). Consistently, the results showed that GITRL expression was upregulated in the mucosal Mϕфof *H. pylori*-positive gastritis patients (**Figure 3A**). We also found that GITRL expression was raised in monocyte-derived macrophages (MDM) infected with *H. pylori* (**Figure 3B**). To further investigate the role of GITRL expressed on Mϕ in the IL-21 production of gastric TFH-like cells, we conducted a co-culture assay with MDM and mucosal CD4^+^T cells to assess the influence of the GITRL/GITR signal on TFH-like cell activation. MDM was pre-incubated with *H. pylori* to upregulate GITRL expression and then co-cultured with gastric CD4^+^T cells in the presence of GITR-blocking Ab, followed by the measurement of IL-21 production. As expected, *H. pylori* primed the MDM-induced IL-21 production by CD4^+^T cells, while GITR blockage inhibited the IL-21 production induced by *H. pylori*-primed MDM (**Figure 3C**). These data indicated the crucial role of the GITRL/GITRL signal in TFH-like cell polarization and IL-21 secretion in *H. pylori*-positive gastritis.

### GITR/GITRL Axis Promotes the Polarization of IL21^+^ TFH-Like Cells Dependent on the STAT3 Signal Pathway

**Figure 4:**
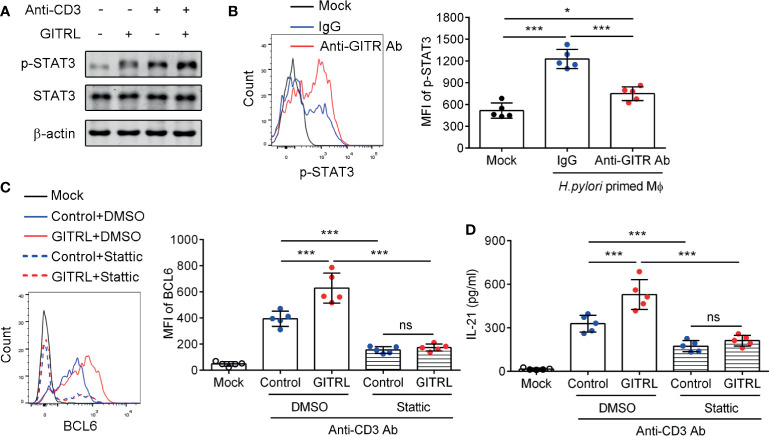
Glucocorticoid-induced tumor necrosis factor receptor (GITR) promoted IL21^+^TFH-like cell polarization dependent on the STAT3 signal pathway in *Helicobacter pylori* infection. **(A)** Gastric CD4^+^T cells were isolated from the gastric mucosa of *H. pylori*-positive patients and treated with CD3 agonist Ab or recombinant GITRL protein for 12 h. Western blot was performed to detect the expression of phosphorylated STAT3 (Ser727) and total STAT3. **(B)** Monocyte-derived macrophages were infected with *H. pylori* (multiplicity of infection = 20) and co-cultured with gastric CD4^+^T cells with the addition of control IgG or anti-GITR-neutralizing Ab. At 12 h later, the phosphorylated level of STAT3 (Ser727) in CD4^+^T cells was determined by flow cytometry. **(C, D)** Sorted gastric CD4^+^T cells were pretreated with stattic or dimethyl sulfoxide for 1 h and stimulated with CD3 agonist Ab or recombinant GITRL protein for 3 days. **(C)** The BCL6 expression in CD4^+^T cells was detected by flow cytometry. **(D)** The IL-21 concentration in the culture supernatant was determined by ELISA. One-way ANOVA was used to compare among three or multiple groups. Data represent mean ± SD from at least three independent experiments. ns, *P* > 0.05; *P < 0.05; ***P < 0.001.

We next explored the effect of GITR on the critical signal pathway which mediates TFH-like cell polarization. The STAT3 signal pathway is essential for classical TFH cell polarization (31). We isolated the gastric CD4 <σπ>+</σπ>T cells and stimulated the cells with recombinant GITRL protein and CD3-activating Ab. The results showed that the phosphorylation of STAT3 of gastric CD4^+^T cells was increased after stimulation with CD3-activating Ab and recombinant GITRL protein. Meanwhile, recombinant GITRL protein synergized with CD3-activating Ab further increased the phosphorylation of STAT3 in gastric CD4^+^T cells compared to the use of CD3-activating Ab alone (**Figure 4A**). Furthermore, to investigate whether GITRL in Mϕф;could induce STAT3 activation in gastric TFH-like cells, we established the co-culture system and found that *H. pylori*-primed Mϕ induced STAT3 phosphorylation in gastric CD4^+^T cells, while GITR blockage reduced STAT3 phosphorylation induced by *H. pylori*-primed Mϕ (**Figure 4B**). We found that classical TFH cell-specific transcription factor BCL6 was increased in gastric IL-21-producing TFH-like cells (**Figure 1C**). To investigate the role of GITR-mediated STAT3 activation in BCL6 expression and IL-21 production, isolated gastric CD4^+^T cells were pretreated with stattic or dimethyl sulfoxide and stimulated with CD3-activating Ab and recombinant GITRL protein. The activation of the GITR signal enhanced the BCL6 expression and IL-21 secretion in gastric CD4^+^T cell stimulated with CD3-activating Ab, whereas inhibition of STAT3 substantially restrained the BCL6 expression and suppressed the IL-21 concentration in the culture supernatant of gastric CD4^+^T cells induced by recombinant GITRL protein (**Figures 4C, D**). These data demonstrated that STAT3 signal played a critical role in GITR-mediated TFH-like cell polarization and IL-21 secretion in *H. pylori*-positive gastritis.

### IL-21^+^TFH-Like Cells Induce B Cell Proliferation in *H. pylori*-Positive Gastritis

**Figure 5:**
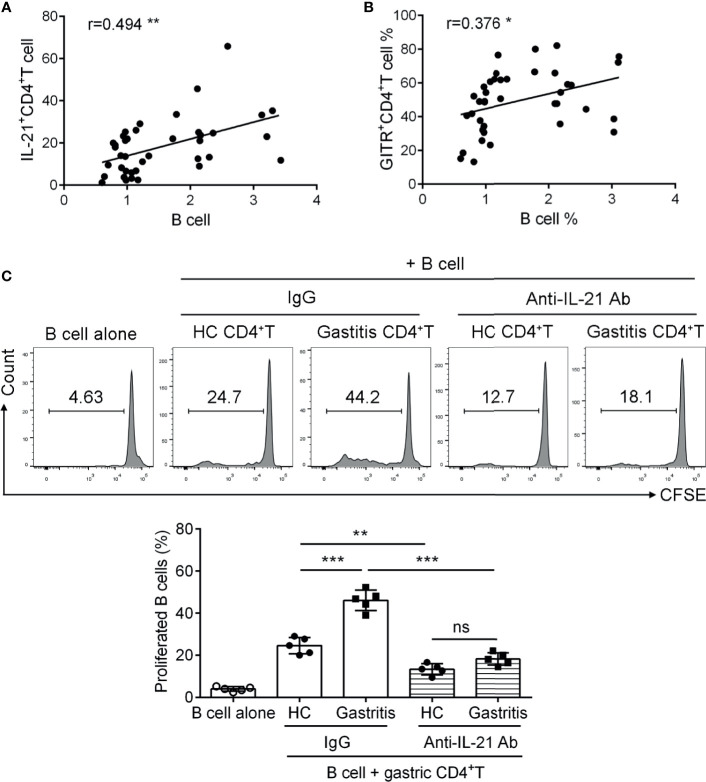
IL-21^+^TFH-like cells induced B cell proliferation in *Helicobacter pylori*-positive gastritis. **(A, B)** The correlations of B cell proportion with **(A)** IL-21 and **(B)** glucocorticoid-induced tumor necrosis factor receptor expression in mucosal CD4^+^T cells of *H. pylori*-positive gastritis patients were analyzed by Spearman correlation analysis (*n* = 39). **(C)** The gastric CD4^+^T cells were respectively isolated from the gastric mucosa of *H. pylori*-positive patients and healthy controls and treated with CD3 Ab or recombinant GITRL protein for 12 h. Then, the gastric CD4^+^T cells were co-cultured with carboxyfluorescein succinimidyl ester-labeled autologous CD19^+^B cells in the presence of IgG or anti-IL-21-neutralizing Ab. At 3 days later, the percentage of proliferated B cells was assessed by flow cytometry. One-way ANOVA was used to compare among three or multiple groups. Data are shown as mean ± SD from at least three independent experiments. ns, *P* > 0.05; *P < 0.05; **P < 0.01; ***P < 0.001.

TFH cells support the proliferation and differentiation of germinal center B cells in lymph nodes (7). To fully elucidate the role of IL-21^+^TFH-like cells in *H. pylori-*positive gastritis, we next explored the regulatory role of IL-21^+^TFH-like cells in B cell function. We firstly analyzed the percentage of gastric CD19^+^B cells as well as the correlation between the percentage of IL-21^+^CD4^+^T cells and CD19^+^B cells in *H. pylori*-positive gastritis patients. Data showed that the percentage of IL-21^+^CD4^+^T cells was positively correlated with the frequency of B cells in the mucosa of *H. pylori*-positive gastritis patients (**Figure 5A**). Meanwhile, a positive correlation was also observed between the percentage of GITR^+^CD4^+^T cells and B cell frequency in the gastric mucosa of gastritis patients (**Figure 5B**). Furthermore, we utilized the co-culture system to investigate the role of IL-21^+^TFH-like cells in gastric B cell response. Gastric mucosal CD4^+^T cells were isolated and pre-treated with CD3-activating Ab and recombinant GITRL protein. The primed CD4^+^T cells were co-cultured with blood CD19^+^B cell labeled with carboxyfluorescein succinimidyl ester in the presence of anti-IL-21-neutralizing Ab or control IgG. The results showed that B cells co-cultured with CD4^+^T cells from *H. pylori*-positive gastritis patients had a higher ratio of proliferation compared to those co-cultured with CD4^+^T cells from healthy controls (**Figure 5C**). Furthermore, the blockage of IL-21 in the co-culture system inhibited B cell proliferation induced by CD4^+^T cells from *H. pylori*-positive gastritis patients (**Figure 5C**). These data indicated that the gastric mucosal TFH-like cells promoted B cell response *via* IL-21 in *H. pylori*-positive gastritis.

### IL-21^+^TFH-Like Cells Promote the Production of Pro-Inflammatory Cytokines and Chemokines as Well as the Secretion of Matrix Metalloproteinases in Human Gastric Epithelial Cells

**Figure 6:**
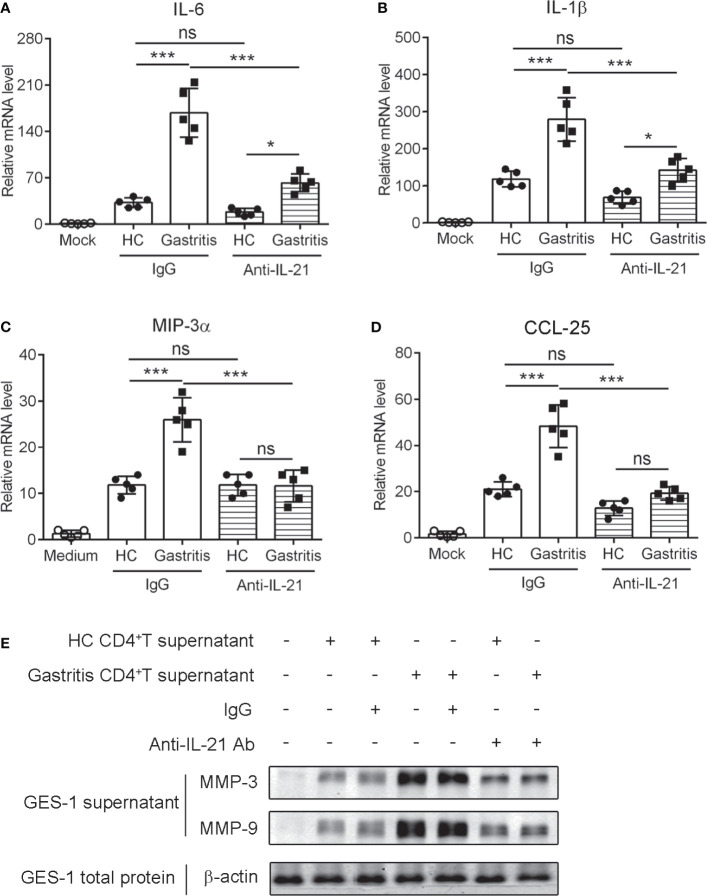
IL-21^+^TFH-like cells induced the expression of pro-inflammatory cytokines and matrix metalloproteinases in human gastric epithelial cells. The gastric CD4^+^T cells were isolated from the gastric mucosa of *Helicobacter pylori*-positive patients and healthy controls and stimulated with CD3 agonist Ab or recombinant GITRL protein. At 3 days later, the culture supernatant was collected. GES-1 cells were treated with 20% culture supernatant of gastric CD4^+^T cells with the addition of control IgG or anti-IL-21-neutralizing Ab for 24 h. The mRNA expressions of IL-6 **(A)**, IL-1ββ; **(B)**, MIP-3αα **(C)**, and CCL-25 **(D)** were determined by real-time PCR. **(E)** MMP-3, MMP-9, and β-actin expression in the culture supernatants or total extracts of GES-1 cells was detected by western blot. One-way ANOVA was used to compare among three or multiple groups. Data are shown as mean ± SD from at least three independent experiments. ns, *P* > 0.05; *P < 0.05; ***P < 0.001.

Human gastric mucosa infected by *H. pylori* triggers the release of inflammatory effectors such as cytokines and chemokines (32). To explore the relevance of IL-21-producing TFH-like cells with *H. pylori*-induced mucosal inflammation, we collected the culture supernatant of gastric CD4^+^T cells stimulated with anti-CD3 agonist Ab or recombinant GITRL protein to treat human gastric epithelial cell line GES-1 cells in the presence of IL-21-neutralizing Ab. The results showed that the culture supernatant of CD4^+^T cell from *H. pylori*-positive gastritis patients induced higher levels of pro-inflammatory cytokine IL-6 and IL-1ββas well as chemokine macrophage inflammatory protein (MIP-3α) and CCL-25 in GES-1 cells compared to the supernatant of CD4^+^T cell from healthy controls (**Figures 6A–D**). Furthermore, the blockage of IL-21 with neutralizing Ab reduced the production of cytokines and chemokines in GES-1 (**Figures 6A–D**). A previous study reported that IL-21 enhanced MMP secretion in human intestinal fibroblasts (14). We demonstrated that the culture supernatant of CD4^+^T cell from *H. pylori*-positive gastritis patients promoted MMP3 and MMP9 secretion of GES-1 cells (**Figure 6E**). Simultaneously, IL-21 blockage decreased MMP3 and MMP9 secretion (**Figure 6E**). Taking these data together, it is indicated that IL-21 produced by mucosal TFH-like cells promoted the production of pro-inflammatory cytokines and chemokines and facilitated the secretion of matrix metalloproteinases in gastric epithelial cells during *H. pylori*-positive gastritis.

## Discussion

In the present study, we explored the regulatory role of the GITR/GITRL axis in facilitating mucosal TFH-like cell activation and IL-21 production in *H. pylori*-positive gastritis patients. Our findings revealed the critical T cell subset contributing to *H. pylori*-mediated gastritis, and we investigated the potentially modulatory mechanisms, which may provide intervening strategies for the clinical management of *H. pylori*-induced gastritis.

GITR is a TNFRSF member widely studied in multiple T cell subsets, such as Treg, Th9, and Th17 (27, 33). Studies indicate that GITR plays a key role in TFH cell differentiation. During chronic lymphocytic choriomeningitis virus (LCMV) infection or collagen-induced arthritis (CIA), GITR expression is upregulated on TFH cells rather than non-TFH cells (34, 35). Meanwhile, the administration of GITR-Fc protein significantly alleviated CIA symptoms and joint damage *via* suppressing TFH cell generation and the production of autoantibodies (34). An impaired TFH generation was observed in GITR knockout mice after chronic LCMV infection (35). Specifically, GITR promotes TFH cell proliferation, thereby favoring antibody production to fight against chronic LCMV infection (35). Taking these results together, the GITR signal positively modulates TFH cell generation. It is reported that TFH cells are induced in the gut mucosa of mice infected with *H. pylori* (36). However, the role of GITR in *H. pylori*-infected gastritis remains unclear. In this study, we found that GITR promoted the polarization of mucosal TFH-like CD4^+^T cells during *H. pylori* infection. GITRL was upregulated in *H. pylori*-infected macrophage and provided a ligand signal to GITR-expressed CD4^+^T cells, which induced TFH-like cell polarization. The STAT3 signal pathway is essential for classical TFH cell differentiation (31), and we demonstrated here that GITR promoted TFH-like cell polarization *via* STAT3 signal in *H. pylori*-positive gastritis.

IL-21 is a characteristic cytokine mainly secreted by TFH cells (6). It is reported to be highly produced in *H. pylori*-infected gastric mucosa and promote the synthesis of gelatinases (18). IL-21 could maintain pro-inflammatory T cell response to drive *H. pylori* infection-induced chronic gastritis (19). Here we identified a subset of tissue-resident CXCR5^-^BTLA^-^PD-1^hi^ TFH-like cells with IL-21 production in the mucosal tissue of *H. pylori*-positive gastritis patients.

In secondary lymphoid tissues, TFH cells exert a crucial function in the formation of the GC and the development of T cell-dependent B cell responses (6). Many clinical studies have indicated that *H. pylori* infection is a key factor of gastric mucosa-associated lymphoid tissue (MALT) lymphoma (37–39). Gastric MALT lymphoma is a B-cell lymphoma that is induced in mucosal barriers. The infection of *H. pylori* is significantly related to lymphoid follicle formation, regarded as the first step in MALT lymphomagenesis of lymphoid expansion (40). Murine model data reveals that *Helicobacter* spp. infection results in pathophysiological alternations that take place at the early phase of MALT lymphomagenesis (41). Dysregulated TFH cells could induce GC B cell outgrowth and lymphomagenesis (42). In this study, we found that gastric TFH-like cell significantly induced B cell proliferation *via* IL-21, which may explain the early lymphomagenesis induced by *H. pylori* infection.

Gut epithelial cells take part in immune response through responding to bacteria, presenting antigens, and secreting cytokines to regulate the activity of mucosal immunity. More importantly, IL-21R is constitutively expressed in intestinal epithelial cells, and its expression is further elevated in the inflamed tissue of UC and CD patients compared with normal controls (16), indicating the probable direct target of IL-21 to intestinal epithelial cells. *In vitro* experiments also show that IL-21 enhances the secretion of MIP-3α by epithelial cell lines (15). MIP-3ααcan bind chemokine receptor CCR6 which is expressed in immune cells, suggesting that MIP-3α might foster the migration and accumulation of immune cells in the gut lamina propria during chronic inflammation.

The physiologic reactions to inflammation-associated damage led to the injuries of the original mucosal structure. The primary players during this process are the myofibroblasts and fibroblasts located in the lamina propria, which produce large amounts of MMPs in inflammatory conditions (43). MMPs are a class of neutral endopeptidases that cleave various components of the extracellular matrix. It has been recently reported that IL-21R is expressed by gut fibroblasts, and the stimulation of IL-21 results in the increased release of MMP-1, MMP-2, MMP-3, and MMP-9 (14). The neutralization of IL-21 significantly reduces MMP secretion when fibroblasts are treated with the culture supernatants of lamina propria mononuclear cells from CD patients, demonstrating the role of IL-21 in MMP-mediated tissue damage.

In conclusion, our data showed that GITR was upregulated on gastric TFH-like cell after *H. pylori* infection. The GITR/GITRL axis enhanced gastric IL-21 production by TFH-like cells. IL-21, produced by mucosal TFH-like cells, induced the B cell proliferation and inflammatory response of gastric epithelial cells. These findings shed light on the function of IL-21-producing TFH-like cells regulated by the GITR/GITRL axis in *H. pylori*-positive gastritis and held the implications for the therapeutic purpose of *H. pylori*-induced gastritis.

## Materials and Methods

### Ethics Statement

This study was approved by the Ethics Committee of Guangzhou Women and Children’s Medical Center, Guangzhou Medical University (approval number 2017021709). The biopsy specimens of *H. pylori*-positive patients and healthy controls were collected from Guangzhou Women and Children’s Medical Center (Guangzhou, China). Informed written consents were obtained from all participants prior to the commencement of the study.

### Subjects

Twenty healthy controls and 39 gastritis patients who appeared with chronic symptoms of peptic disease, including dyspepsia and recurrent abdominal discomfort or pain, were enrolled in this study. The exclusive criteria included a history of acute onset of symptoms, acute or chronic vomiting, and the use of antibiotic, antacid, H2 blockers, proton pump inhibitors, bismuth-containing compounds, or non-steroidal anti-inflammatory drugs within the preceding 4 weeks. Biopsy specimens were obtained from the patients with indications for gastroscopy examination. The samples were stained with Giemsa dye to observe *H. pylori* under a light microscopy. All 39 patients were diagnosed as cases of *H. pylori*-positive gastritis with pathological changes of the mucosa, whereas 20 healthy controls are *H. pylori*-negative and with normal mucosa (see **Table 1**).

### Preparation of Mucosal Mononuclear Cells

Mucosal mononuclear cells were isolated from the gastric mucosa biopsy of the patients as previously described (4). Briefly, biopsy specimens were digested with shaking at 37°C for 45 min. The digestion solution is composed of RPMI 1640 medium (Gibco) containing 10% collagenase D (100 µg/ml; Sigma-Aldrich, St. Louis, MO, USA) and 1% DNase I (10 µg/ml; Thermo Fisher Scientific, Waltham, MA, USA). After complete digestion, the cells were filtered *via* a 70-µm cell filter and collected by centrifuging at 1,500 rpm. Then, the cells were washed and suspended with RPMI 1640 for the following experiments, including cell sorting, co-culture assay, flow cytometry, and immunofluorescence staining (see below).

### Cell Sorting

CD4^+^T cells were isolated from mucosal mononuclear cells by positive selection with magnetic cell sorting system of BD Biosciences. The purity of sorted CD4^+^T cells was determined as >95% by flow cytometry, gated on CD3^+^CD4^+^T cells.

CD14^+^ monocytes were sorted from human peripheral blood mononuclear cells by positive selection with human CD14 magnetic particles (BD). MDM was generated from monocytes as previously described (44). In brief, CD14^+^ monocytes were differentiated into MDM in the presence of 10 ng/µl macrophage colony-stimulating factor for 5 days.

### Co-Culture Assay

MDM were pre-incubated with *H. pylori* (multiplicity of infection, MOI = 20) for 24 h. After the incubation, the Mϕ were irradiated, fully washed, and co-cultured with gastric CD4^+^T cells at a ratio of 1:5 in a 96-well flat-bottomed plate (2 × 10^4^ Mϕ and 1 × 10^5^ CD4^+^T cells per well). The percentage of IL-21-producing CD4^+^T cells was calculated by gating on live CD4^+^T cells.

To evaluate the effect of GITR on the IL-21 production of CD4^+^T cells, recombinant human GITR ligand (10 µg/ml, BioLegend, Product #559206), blocking antibody (Ab) of GITR (10 µg/ml, Clone 621, BioLegend), and agonist Ab of CD3 (10 µg/ml, Clone UCHT1, BD) were added to the co-culture system. At 3 days later, the secretion of IL-21 by CD4^+^T cells was determined by flow cytometry.

### Flow Cytometry Analysis

The procedure of cell staining and flow cytometry was previously described (5). For intracellular staining of IL-21, gastric CD4^+^T cells were restimulated with 1 μg/ml anti-CD3 Ab (Clone UCHT1, BD), 1 μg/ml anti-CD28 Ab (Clone CD28.2, BD), and 3 μg/ml brefeldinA (eBioscience, CA, USA) for 12 h. The intracellular fixation/permeabilization buffer set (eBioscience, CA, USA) was used in IL-21 staining. Flow cytometric detection was performed with FACS Canto II (BD, NJ, USA). Data was analyzed by the FlowJo software (Tree Star). The anti-human antibodies used in this study were purchased from Biolegend, BD Biosciences, or Miltenyi Biotec, namely: CD3 (Clone UCHT1, BioLegend), CD4 (Clone OKT4, BioLegend), GITR (Clone 108-17, BioLegend), GITRL (Clone REA841, Miltenyi), IL-21 (Clone 3A3-N2.1, BD), BCL6 (Clone 7D1, BioLegend), STAT3 Phospho (Ser727) (Clone A16089B, BioLegend), BTLA (Clone MIH26, BioLegend), CXCR5 (Clone J252D4, BioLegend), and PD-1 (Clone EH12.2H7, BioLegend).

### Immunofluorescence Staining and Confocal Microscopy

Paraffin-embedded samples were cut into 5-μm slices, and immunohistochemistry was conducted as previously described (4). In brief, the mucosa was fixed with paraformaldehyde, followed by antigen retrieval with the EDTA antigen retrieval solution (Servicebio). The processed samples were blocked with 5% bovine serum albumin (BSA) and subsequently incubated with CD4 (Clone RPA-T4, BioLegend) and IL-21 (Abbiotec, Product #253397) Abs, followed by staining of secondary Abs Alexa Fluor^®^ 488 (Thermo, Product #A-11034) and Alexa Fluor^®^ 594 (Thermo, Product #A-11005) as well as DAPI (Thermo, Product #A-11034) before the detection. Fluorescence images were captured under a ZEISS IMAGER A1 fluorescence microscope (CARL ZEISS).

### Western Blot

The cells were collected, washed three times with cold phosphate-buffered saline (PBS, pH 7.4, Invitrogen), and lysed by RIPA lysis buffer (Sangon Biotech, C500005) containing 1% (v/v) protease inhibitor cocktail (Merck, P8340), 1 mM phenylmethylsulfonyl fluoride (Merck, 52332), and 1 mM DTT (Merck, DTT-RO). Equivalent amounts (20 μg) of cell lysates were separated by SDS-PAGE and then transferred to polyvinylidene difluoride membrane. The membranes were blocked in PBS-Tween20 (pH 7.4, 0.5% Tween20) containing 5% BSA for 1 h at room temperature (RT) and then incubated overnight with primary antibodies at 4°C. On the next day, the membranes were incubated with appropriate horseradish peroxidase-conjugated secondary antibodies at RT for 1 h, followed by visualization with an ECL kit (KeyGEN, Nanjing, China) based on the recommendations of the manufacturer.

### Real-Time PCR

Gastric mucosa was collected and homogenized by bead-milling as previously described (4). The cells were lysed, and total RNA was extracted with TRIzol reagent (Invitrogen) (refer to the instructions of the manufacturer). The synthesis of first-strand cDNA was performed by Synthesis Kit (Ref). The expression level of the indicated genes (IL-6, IL-1β, MIP-3α, CCL-25, and β-actin) was examined by real-time PCR amplification using SYBR Green Master Mix kit (Invitrogen) and detected with the Bio-Rad CFX96 real-time detection system. The mRNA expression of the target genes relative to β-actin was calculated according to the following formula: relative mRNA expression = 2^CT value (actin–target gene)^.

### ELISA

The concentration of IL-21was detected by Human IL-21 ELISA kit (MultiSciences, Product #70-EK121HS) according to the manufacturer’s instructions. For IL-21 release of CD4^+^T cells, gastric CD4^+^T cells were isolated and restimulated with 1 µg/ml CD3 Ab and 10 µg/ml recombinant GITRL protein for 3 days, followed by the collection of the culture supernatant for ELISA assay.

### Statistical Analysis

Statistical analysis was performed with GraphPad Prism 5.0 (GraphPad Software, San Diego, CA). Significance was analyzed by chi-square test, Spearman correlation analysis, one-way analysis of variance, or unpaired Student’s *t*-tests. Data are shown as mean ± SD unless otherwise indicated. *P*-value less than 0.05 is considered significant.

## Data Availability Statement

The original contributions presented in the study are included in the article/supplementary material. Further inquiries can be directed to the corresponding author.

## Ethics Statement

This study was approved by the Ethics Committee of Guangzhou Women and Children’s Medical Center, Guangzhou Medical University (approval number 2017021709). The patients/participants provided their written informed consent to participate in this study.

## Author Contributions

SM and YW initiated and designed the research. SM, YW, and SG wrote the manuscript with the help of other co-authors. SM, HY, and XL performed the experiments and analyzed and/or interpreted the results. YW, GZ, and SG were in charge of patient care and contributed to the discussion of the results. All authors contributed to the article and approved the submitted version.

## Funding

This work was supported by grants from the National Natural Science Foundation of China (81770552, 81801571 and 82102249), the China Postdoctoral Science Foundation (2020T130131), the National Key Research and Development Plan (2019YFC0840602), the Guangdong Scientific and Technological Foundation (2020B1111170014 and 2019A1515110055), and the Shenzhen Scientific and Technological Foundation (KCXFZ202002011007083 and JCYJ20180228162321234).

## Conflict of Interest

The authors declare that the research was conducted in the absence of any commercial or financial relationships that could be construed as a potential conflict of interest.

## Publisher’s Note

All claims expressed in this article are solely those of the authors and do not necessarily represent those of their affiliated organizations, or those of the publisher, the editors and the reviewers. Any product that may be evaluated in this article, or claim that may be made by its manufacturer, is not guaranteed or endorsed by the publisher.
